# Splenic cysts, many questions are yet to be answered: a case report

**DOI:** 10.4076/1757-1626-2-8474

**Published:** 2009-07-10

**Authors:** Abu-Ella Amr

**Affiliations:** Department of General Surgery, El Sahel Educational HospitalCairoEgypt

## Abstract

We present two patients with splenic cysts. The first is a female 29-years-old who presented with upper abdominal discomfort of one-year duration. Imaging revealed a huge splenic cyst. Splenectomy was undertaken. Pathological examination revealed a lymphatic cyst. The second was an 18-years-old female who presented to the emergency department with a ruptured splenic cyst. Splenectomy was undertaken, it proved to be a simple cyst.

## Introduction

Splenic cysts are an uncommon encounter in surgical practice and less than 1000 cases have been reported [1,2]. Most patients with splenic cysts experience minor, nonspecific symptoms related to the mass effect of the cyst. The diagnosis is made by taking a thorough patient history, conducting a physical examination, and evaluating ultrasonography and CT scan findings. Rupture, hemorrhage, and infection, which may be life-threatening, have been reported [3].

## Case presentation

### Case report 1

**Figure 1.**
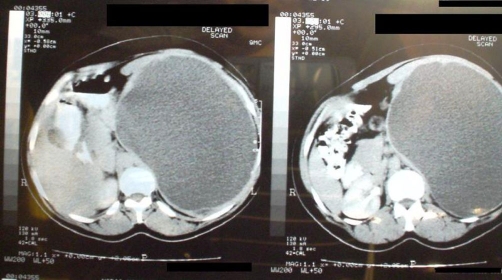
CT scan showing large splenic cyst reaching midline.

An Egyptian 29-years-old female, with BMI of 25, accountant, non-smoker, presented with upper abdominal discomfort and dull pain in her left loin of one year duration. The patient had no history of trauma or schistosomiasis, which is a common condition in Egypt. Examination revealed huge splenomegaly. Spleen was broad and crossing midline. CT scan (Figure 1) showed a huge splenic cyst, which has 2 components; an upper multilocular component and a lower unilocular one. No signs of hydatid infestation on CT. The left kidney did not show on CT contrast enhancement. Laboratory tests were unremarkable, serological tests were negative for hydatid.

**Figure 2.**
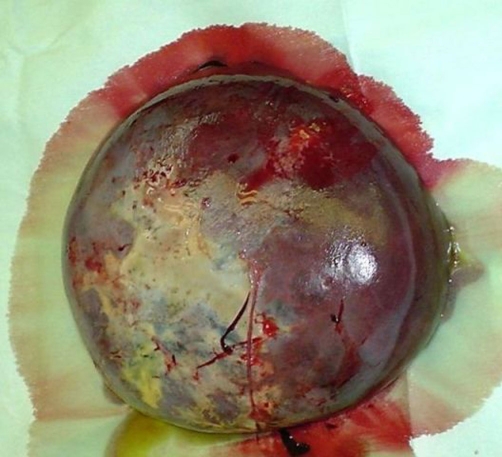
Splenectomy specimen showing the large size and fibrinous adhesions.

Exploratory laparotomy was undertaken. The spleen was huge (Figure 2) and toughly adherent to the posterior abdominal wall. It was almost completely replaced with the cyst but the wall or splenic rim of tissue was thick. The left kidney was remarkably displaced downwards. Tortuous dilated veins were found at the hilum. After freeing the adhesions with blunt dissection delivery of the spleen was done, control of the splenic artery and vein through lesser sac secured further dissection, mobilization and extraction. The patient had an uneventful postoperative course.

Pathology report showed a lymphatic cyst with multilocular components.

### Case report 2

**Figure 3.**
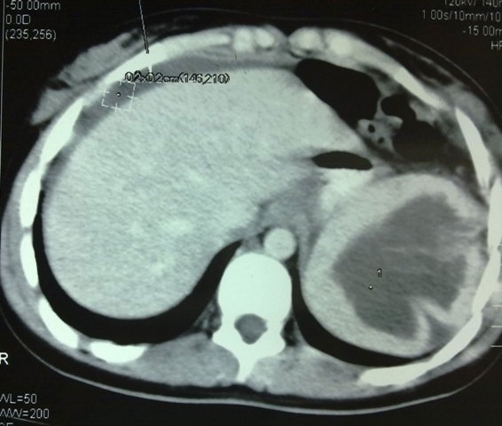
CT scan showing the ruptured cyst.

An Egyptian female student 18-years-old presented to emergency department with acute abdomen of few hours duration. Examination revealed a vitally stable patient with generalized abdominal tenderness. Lab results showed mild leukocytosis, normal HB%. Abdominal ultrasound showed suspected splenic tear, with free intraperitoneal free fluid. CT revealed a splenic cyst? (Figure 3) ruptured. Serological test for hydatid was negative.

**Figure 4.**
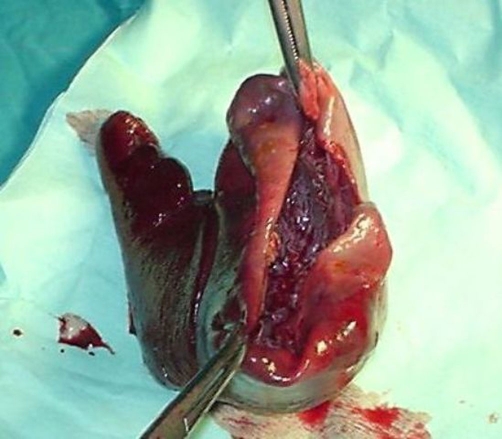
Spleen showing the upper pole cyst.

We decided laparotomy due to presence of loculations on CT. Laparotomy and splenectomy were undertaken. Pathological examination revealed a simple cyst (Figure 4).

## Discussion

The treatment of splenic cysts is a difficult challenge to surgeons [4]. Not surprisingly with the classification, diagnostic modalities and treatment guidelines are far from being uniform or clear.

Three classification systems are present Fowler, Martin and Morgenstern [5].

Discovery of the cyst is easy with imaging modalities but the true nature and pathology of the cyst is not always possible to determine preoperatively [6]. Since the pathology dictates management, decision making for the ultimate management is still personal.

Preoperative aspiration of the fluid for biochemical and cellular examination is debatable. Some recommends it routinely [4] while others see it is contraindicated and hazardous [2].

A wide range of treatment modalities have been described for symptomatic or very large cysts of the spleen. Nonoperative measures, such as observation, have been recommended for asymptomatic cysts smaller than 5 cm. The natural history of these small cysts is largely unknown, but if the imaging characteristics reveal regularity of the cyst wall, absence of a solid component, and a typical round shape, there is no indication for cyst removal. Spontaneous resolution of traumatic pseudocysts can occur. Surgical treatment usually is recommended for symptomatic patients or for those with cysts larger than 5 cm [2].

Percutaneous aspiration of the cyst has been described as a definitive treatment, but this option often leads to recurrence. Chemical agents, such as alcohol or tetracycline, have been percutaneously injected into the cyst cavity after aspiration in an attempt to collapse the cyst wall, promote fibrosis, and prevent re-accumulation; however, recurrences were reported [7,8]. Therefore, percutaneous aspiration with or without sclerosis should not be considered a definitive treatment and may complicate subsequent surgical management.

Total splenectomy used to be the gold standard for splenic cysts. However, due to the increasing awareness of the immunologic function of the spleen, organ-preserving techniques were developed to avoid the rare but life-threatening risk of overwhelming post-splenectomy sepsis. These salvage procedures ranged from cyst excision with partial splenectomy to cyst marsupialization with partial cyst wall excision [7].

With the advent of advanced laparoscopy, previously standard open operations for the treatment of nonparasitic splenic cysts have been undertaken using a minimally invasive approach [8].

Despite such successes, it is important to note that laparoscopic splenectomy still incurs a risk of post-splenectomy sepsis and that laparoscopic partial splenectomy is a technically challenging operation with a longer operative time and the potential for greater blood loss [6,9].

## Conclusion

A standardized approach for diagnosis, classification and management of splenic cysts is waited for.
